# The Contribution of Individual Psychological Features to the Determination of the Phenomenon of Work Alienation

**DOI:** 10.3390/bs10010034

**Published:** 2020-01-15

**Authors:** Leonid V. Vinokurov, Anastasiia A. Kozhina

**Affiliations:** 1 Division of the System Research of the Formation of Sports Mastery, Saint-Petersburg Scientific-Research Institute for Physical Culture, 191040 Saint.-Petersburg, Russia; leon_1205@mail.ru; 2 Department of Psychology of Professional Activity, The Herzen State Pedagogical University of Russia; 191186 Saint.-Petersburg, Russia

**Keywords:** work alienation, personality traits, psychological needs, need for autonomy, need for competence, need for relatedness

## Abstract

Using the questionnaire method on a sample of 92 Russian-speaking employees of the “person-person” professions type, the relationship of work alienation with personal characteristics was investigated. It was found that work alienation has associations with some personality traits. Also, the satisfaction of basic psychological needs at work is a significant predictor of work alienation. The study proved that work alienation is a relatively flexible construct for changes in the workplace that relate to autonomy, competence, and social relations. The results of the research can be used as an empirical justification of the model of the phenomenon as well as in the development of a differentiated approach to the prevention and intervention of work alienation, depending on the personality characteristics of employees.

## 1. Introduction

This paper is dedicated to work alienation, a phenomenon which represents “a distance or separation from work, its context or itself” [1]. It represents subjectively endured state connected with destruction of interpersonal communications, which is expressed in perception by the subject of work of helplessness, senselessness, and self-alienation.

The construct of alienation has a long history in philosophy and sociological science, although as subject of psychological field, particularly on the Russian-language sample, it remains a little studied. Previous studies have shown a link between work alienation and organizational variables as work performance, organizational commitment, job satisfaction, job involvement, and organizational identification [2]. Another observation in the context of work alienation is the increase of burn out, deviant behavior, and alcohol use [1]. This all speaks about the high importance of this problem. Nevertheless, the question of psychological predictors and especially personality factors of work alienation was studied fragmentary and inconsistently.

One of the most influential theories of work alienation belongs to Kunungo. He viewed work alienation “as a generalized cognitive state (or belief) of psychological separation from work insofar as work is perceived to lack potentiality for satisfying one’s salient needs and expectations” [3] (p. 131). A number of other researchers have also suggested that if employees experienced low satisfaction or frustration of relevant psychological needs in a job context, they become alienated [1,4,5]. Similar to previous theories that applied a motivational approach [1,3,4,5], we assume that work alienation is a complex state in which a person is not interested in their work, does not like it, and considers it to be replaceable. This is because the concrete occupational activities, the organization and the relationships to other employees do not fulfill their intrinsic needs. We suggest that basic needs (autonomy, belongingness, competence) formulated in the Self-Determination Theory (SDT) [6] play an essential role in the emergence of alienation.

According to SDT of Deci and Ryan [6], the satisfaction of the intrinsic needs for competence, autonomy, and social relatedness accounts for the emergence of intrinsic motivation, which is consequently believed to result in the opposite of alienation. [3,4,7,8,9]. Deci and Ryan define the need for autonomy as the desire for freedom and doing things on one’s own volition. The need for autonomy can be satisfied in an organizational context by goal setting, decision making, work planning, etc. [6]. For example, Farahbod and colleagues took the lack of employee participation in decision making into account for the emergence of work alienation as well as routine work [10]. The need for competence refers to an inner motivation to be effective and to attain a valued outcome. In the framework of concrete job, this need can be satisfied through measures such as positive feedback. The third essential need formulated by the SDT is the need for relatedness (belongingness). It refers to the desire to feel connected to others—to love and care, and to be loved and cared for [6]. Optimizing employee communication processes, transformational leadership, and improving the social atmosphere in the workplace can contribute to the satisfaction of this psychological need. Ground on the SDT, we suggest that frustration of basic psychological intrinsic needs in a workplace can predict work alienation:

**Hypothesis** **1.** *Satisfaction of basic psychological needs at work negatively predicts work alienation*.

**Hypothesis** **1a.** *Satisfaction of the need for autonomy at work negatively predicts work alienation*.

**Hypothesis** **1b.** *Satisfaction of the need for competence at work negatively predicts work alienation*.

**Hypothesis** **1c.** *Satisfaction of the need for relatedness at work negatively predicts work alienation*.

In order to know how to efficiently design interventions, it is necessary to understand to what extent an alienated person can be “cured” from the alienation, or to what extent it is an unchangeable attribute of the person. Concerning temporal perspective, work alienation was studied only in one research. Zeller, Neal, and Groat [11] examined work alienation in longitudinal study over eight years. Construct of work alienation was measured through variables of powerlessness, isolation, normlessness, and meaninglessness separately and observed a high stability in all scales over the eight years, period. At this juncture, it needs to be stated that their items were not solely work alienation related, but defined alienation in broader contexts. The study also had limitations regarding the representativeness of the sample and therefore still leaves the question of temporal variability or stability of work alienation open. 

Contextual factors and such job characteristics as organizational structure, occupational prestige, promotional opportunity, and work routineness vary over job contexts and are therefore considered as variable [4]. Personality traits and factors such as age, sex, marital status, and education can be regarded as stable over work contexts. In order to draw conclusions about the variability of alienation, we will try to define relationship of work alienation with some personality characteristics of employees. A review of existing research on the topic of work alienation provides little knowledge about the relationship of this construct with the personal characteristics of the subjects of labor. Osin [12] investigated the relationship between alienation and various personal factors/personality traits. Work alienation was analyzed as an expression of alienation in one of the spheres of life, along with society, relationships, family, and the self. It was found that work alienation is negatively associated with subjective well-being (life satisfaction and happiness), life meaning, psychological well-being, hardiness, sense of coherence, self-determination, and internal locus of control. However, based on prior studies of the relationship of related to work alienation constructs and personality traits, it is possible to make the assumption of the presence of those concerning alienation. Bozionelos [13] found out that there is a significant but not strong or extensive relationship between work involvement and the Big Five of personality traits. According to meta-analysis, job satisfaction, a similar concept to work alienation, has negative correlation with neuroticism and positive correlation with extraversion [14]. Similar to the above mentioned literature, we suggest that some personality traits responsible for social adaptation and regulation of behavior may play a role in the subjective perception of work alienation.

These assumptions are derived in general hypothesis:

**Hypothesis** **2.** *There is a significant correlation between work alienation and some personality traits.*

**Hypothesis** **2a.** *Work alienation is positively associated with neuroticism.*

Alienation as a psychological state is expressed in powerlessness and helplessness, which is similar to the concept of depression. It is also reported in the literature that work alienation is directly associated with depression disorder [15]. Depression can be defined as personality trait that refers to signs of despair, unhappiness, and oppression in an emotional state, in behavior, in relations to oneself, and in relations to the social environment. We assume that depressiveness as personality trait has association with work alienation:

**Hypothesis** **2b.** *Work alienation is positively associated with depression.*

Sociability as a personality trait is understood as potential opportunities and real manifestations of social activity. Since the work alienation is considered by many authors as a complex and multi-component construct related to social isolation and the violation of communication processes [16,17], the sociability would be probably negatively associated with alienation:

**Hypothesis** **2c.** *Work alienation is negatively associated with sociability.*

Based on the same assumption, work alienation can be associated with reactive aggressiveness, which is a personality trait that refers to the tendency of the aggressive relation toward a social environment and the expressed desire for dominance:

**Hypothesis** **2d.** *Work alienation is positively associated with reactive aggressiveness.*

According to research, another personality trait that may have a relationship with work alienation is introversion. Introversion relates to solitary behavior, a focus on the inner world and not to the outside world or communication with other people. Based on the study of similar to work alienation construct, namely job satisfaction, we assume that work alienation is associated with introversion:

**Hypothesis** **2e.** *Work alienation is positively associated with introversion.*

Emotional lability is another personality trait that may be related to work alienation. This personality trait is manifested in the instability of the emotional state, increased excitability, and insufficient self-regulation. In literature, evidence was found that suggests emotional variability has a negative effect on job satisfaction [18]. These results leads us to the following hypothesis:

**Hypothesis** **2f.** *Work alienation is positively associated with emotional lability.*

## 2. Materials and Methods

We assessed our data through a questionnaire, which was sent to several state Russian companies. Employees of the “person-person” professions type were invited to participate in the study. Professions of this type are characterized by the fact that the content of labor consist regular and intensive interaction with people [19]. Participants were invited through local managers and received a small material reward for participating in the study. The questionnaire was available in a paper-pencil format. The measurement started in the first week of September 2018. The data was collected anonymously and the participants were informed via an introductory text about the anonymity and voluntariness of their participation. Participants signed an informed consent form. 

Work alienation was measured by AEA-kJ: dt.: “Fragebogen zur arbeitsbezogenen Entfremdung und Aneignung – konkreter Job”; eng.: “Questionnaire of work alienation – concrete job” of Aigner et al. [20], adapted for the Russian-language sample by Vinokurov and Kozhina. The questionnaire consisted of a five item short scale on a 6-point Likert scale (1 = strongly disagree, 6 = strongly agree) addressing the experience of alienation at the job level. An example item is: “I like to think about my job”. Cronbach’s alpha for this scale is 0.80, which indicates good reliability. 

Personal characteristics were studied using FPI: dt.: “Freiburger Persönlichkeitsinventar - B”: eng.: “Freiburg Personality Inventory”- Option «B» [21] in adaptation of Krylov and Ronginskaya [22]. The questionnaire is designed to diagnose personality traits that are essential for the process of social adaptation and behavioral regulation. It includes 12 scales (neuroticism, spontaneous aggressiveness, depression, irritability, sociability, stress tolerance, reactive aggressiveness, shyness, openness, extraversion-introversion, emotional lability, and masculinity-feminism), with a total amount of 114 questions. The Items are with “right” or “not right” to answer. Cronbach’s alpha of scales are between 0.79 and 0.83.

The Work-related basic need satisfaction scale [23] in adaptation of Osin was also applied [24]. The questionnaire consisted of six items for competence, seven items for autonomy, and eight items for relatedness (belongingness) which were rated on a 7-point Likert scale (1 = completely wrong, 7 = absolutely right). All scales show good reliability values, since Cronbach’s alpha of scales are between 0.80 and 0.84

The questionnaire was finished by 100 participants. After purging the sample, because of missing assignment data, 92 participants remained. These 92 represent our present sample. The gender distribution was 34% male and 66% female, and the average age was 41.39 years (SD = 12.3). Participants were employed in different industries (66.3% in bank, 16.3% in education, 10.8% in consulting, and 6.5% in psychology). 77.1% of them were account managers and 22.8% were consultants. The mean length of service in the current workplace was 3.2 years (range > 1 < 15). More than 90% of participants had a working week of 36–40 h. All participants had permanent contracts of employment.

All calculations of this study were conducted with SPSS 22. At the beginning of the evaluation, reliability analyses was carried out to check the internal consistency of the scales. The results of the reliability analysis showed that the Cronbach’s alpha of all scales are between 0.79 and 0.84, and therefore have a good internal consistency. To examine the effects of satisfaction of basic psychological needs on work alienation, we performed linear regression analysis. To evaluate the relationship between personality traits and work alienation, Pearson correlations were conducted. 

## 3. Results

### 3.1. Satisfaction of Basic Psychological Needs at Work

The results of the regression analysis presented in Table 1 show that 28% of the variance of variable work alienation can be explained by the influence of independent variable of satisfaction of need for autonomy. Satisfaction of the need for autonomy at work negatively predicts work alienation. 

As can be seen in Table 2, variable satisfaction of need for competence negatively predicts work alienation and explains 29% of the variance of work alienation.

Table 3 illustrates that 31% of the variance of variable work alienation can be explained by the influence of independent variable of satisfaction of need for relatedness. Thus, satisfaction of the need for relatedness negatively predicts work alienation.

### 3.2. Personality Traits

The results of Pearson correlations of Questionnaire of work alienation–concrete job and FPI scales can be seen in Table 4. 

The results showed a positive association between work alienation and such personality characteristic as depression (r = 0.43, *p* < 0.001). The scale sociability showed a negative association with work alienation (r = −0.42, *p* < 0.001). The scale reactive aggressiveness also showed a positive association with work alienation (r = 0.32, *p* < 0.001). There is a positive association between introversion and work alienation (r = 0.34, *p* < 0.001). There is a positive association between emotional lability on the one hand and work alienation on the other hand (r = 0.38, *p* < 0.001). The results of Pearson correlations showed no significant values between work alienation and other scales of FPI questionnaire: neuroticism, spontaneous aggressiveness, irritability, stress tolerance, shyness, openness, and masculinity-feminism.

## 4. Discussion

The aim of the present study was to shed light on certain potential psychological predictors of work alienation. 

In line with Kanungo [3], in our first hypothesis, we expected that the frustration of basic psychological needs at work would predict work alienation. The result of regression analysis supports that work alienation is negatively predicted by satisfaction for needs for autonomy, competence, and relatedness. Therefore, hypotheses 1a, 1b, and 1c can be confirmed. The satisfaction of these needs is possible within conditions and the content of concrete work. Therefore, depending on these factors, alienation from work can be considered as a rather mobile construct. Thus, this complements the previous literature, which considers the alienation of work as a motivational phenomenon and argues that an organization should offer working conditions that allow employees to act self-determined, to foster need satisfaction and therefore prevent work alienation.

The second general hypothesis, which was that work alienation would have association with some personality traits was confirmed for five out of seven scales of FPI proposed for hypotheses. 

The Hypothesis 2a cannot be confirmed as no significant correlation was found between neuroticism and work alienation. These results differ from the conclusions in which job satisfaction has negative correlations with neuroticism [14]. The discrepancy between these results may be due to the difference in the content of these psychological constructs. Neuroticism shows a tendency to experience feelings of anxiety, fear, jealousy, and envy, while alienation is more associated with feelings of powerlessness, meaninglessness, and depression. 

Previous literature has already shown that alienation leads to a state of depression [1]. In this study, however, it was shown that work alienation is associated with depressiveness as a personality trait. Hypothesis 2b can be confirmed.

Work alienation is also negatively associated with sociability and positively with reactive aggressiveness. Therefore, Hypotheses 2c and 1d can be confirmed. Both sociability and reactive aggressiveness are related to the sphere of social activity. Thus, alienated employees express little social interactions and the need for communication, as well as aggressive behavior in relation to the social environment. These results indicate that violations in the communicative sphere are related to the content of the phenomenon of alienation. 

It was also found that work alienation has association with introversion, so Hypothesis 2e can be confirmed. Bozionelos [13] and also Judge, Heller and Mount [14] came to similar conclusions.

In addition, the alienation from work is associated with personality traits such as emotional lability, which consequently confirms the Hypothesis 2f. It means that employees who experience alienation tend to have a worse psychological well-being. These results are consistent with previous research findings [1,2]. 

These findings allow the development of a differentiated approach to the prevention and intervention of work alienation, depending on the personality traits of employees. Based on the fact that personality traits represent stable psychological constructs, we can conclude that in constant and stable environmental conditions, work alienation is also a relatively steady and stable over time construct.

We also need to mention the limitations of our study. Regarding the sample size, our sample of 92 participants was rather small. Our aim was more than 100 participants, but the feedback was low. This could be due to a lack of motivation on side of the participants, since completing the questionnaire took approximately 30 minutes which is rather long. Regarding the distribution of sexes, the majority of participants were female (66%). Nevertheless, these data are close to distribution in the general population. Employees of the “person-person” professions type were involved in the study (education, consulting, psychology, etc.), which are typical for women. For future research, it would be necessary to create the study with a sample which is more balanced by the gender criterion and more homogeneous regarding professional specialization.

As mentioned above, it was found that significant correlations between work alienation and some personality traits exist. On this basis, future studies should test the moderator and mediator effects of various personality traits on work alienation. Since depression, sociability, reactive aggressiveness, introversion, and emotional lability has associations with work alienation, it might be a promising approach to examine preventions that are based on these personality traits. As our research follows assumptions that work alienation represents a relative stable construct under constant environmental conditions, future research longitudinal studies over a longer amount of time are needed. 

Regarding practical implications, our results may mean new positive prospects for employers as they lead to the assumption that alienation can be altered by changes in the working environment. This opens space for improving employee satisfaction as, for example, job enlargement, job enrichment, or the implementation of transformational leadership. As mentioned earlier, research using longitudinal design is required to confirm these propositions in the future.

## Figures and Tables

**Table 1:** Effect of satisfaction of need for autonomy on work alienation.

Predictor	B	SE	β	R²
Autonomy	0.82	0.72	0.45**	0.28 **

** *p* < 0.01.

**Table 2:** Effect of satisfaction of need for competence on work alienation.

Predictor	B	SE	β	R²
Competence	1.26	0.48	0.53**	0.29 **

** *p* < 0.01.

**Table 3:** Effects of satisfaction of need for relatedness on work alienation.

Predictor	B	SE	β	R²
Relatedness	0.60	0.17	0.57**	0.31 **

** *p* < 0.01.

**Table 4:** Pearson correlation values of Questionnaire of work alienation–concrete job and FPI scales.

	WA-j
FPI- neuroticism	0.21
FPI - spontaneous aggressiveness	0.06
FPI - depression	0.43 **
FPI - irritability	0.15
FPI - sociability	−0.42 **
FPI - stress tolerance	−0.08
FPI - reactive aggressiveness	0.32 **
FPI - shyness	0.14
FPI - openness	−0.24
FPI - introversion	0.34 **
FPI - emotional lability	0.38 **
FPI - masculinity-feminism	0.11

** *p* < 0.01. WA-j = work alienation on the level of concrete job.
